# Parkinson’s Disease Severity at 3 Years Can Be Predicted from Non-Motor Symptoms at Baseline

**DOI:** 10.3389/fneur.2017.00551

**Published:** 2017-10-30

**Authors:** Alba Ayala, José Matías Triviño-Juárez, Maria João Forjaz, Carmen Rodríguez-Blázquez, José-Manuel Rojo-Abuin, Pablo Martínez-Martín

**Affiliations:** ^1^Centre for Human and Social Sciences, Spanish Scientific Research Council (CCHS, CSIC) and Red de Investigación en Servicios de Salud en Enfermedades Crónicas (REDISSEC), Madrid, Spain; ^2^West Health District, Primary Care Center Francia, Madrid Health Service, Madrid, Spain; ^3^Epidemiology and Biostatistics Department, National School of Public Health, Institute of Health Carlos III and Red de Investigación en Servicios de Salud en Enfermedades Crónicas (REDISSEC), Madrid, Spain; ^4^National Center of Epidemiology and Centro de Investigación Biomédica en Red sobre Enfermedades Neurodegenerativas (CIBERNED), Institute of Health Carlos III, Madrid, Spain; ^5^Institute of Economics, Geography and Demography, Centre for Human and Social Sciences, Spanish National Research Council (CSIC), Madrid, Spain

**Keywords:** Parkinson’s disease, disease global severity, predictive model, multilevel analysis, multiple imputation

## Abstract

**Objective:**

The aim of this study is to present a predictive model of Parkinson’s disease (PD) global severity, measured with the Clinical Impression of Severity Index for Parkinson’s Disease (CISI-PD).

**Methods:**

This is an observational, longitudinal study with annual follow-up assessments over 3 years (four time points). A multilevel analysis and multiple imputation techniques were performed to generate a predictive model that estimates changes in the CISI-PD at 1, 2, and 3 years.

**Results:**

The clinical state of patients (CISI-PD) significantly worsened in the 3-year follow-up. However, this change was of small magnitude (effect size: 0.44). The following baseline variables were significant predictors of the global severity change: baseline global severity of disease, levodopa equivalent dose, depression and anxiety symptoms, autonomic dysfunction, and cognitive state. The goodness-of-fit of the model was adequate, and the sensitive analysis showed that the data imputation method applied was suitable.

**Conclusion:**

Disease progression depends more on the individual’s baseline characteristics than on the 3-year time period. Results may contribute to a better understanding of the evolution of PD including the non-motor manifestations of the disease.

## Introduction

Parkinson’s disease (PD) is a neurodegenerative, chronic, progressively disabling, and incurable disease, which occurs in about 2% of the population over 65 years old (1). From a clinical perspective, it is characterized by the onset of motor symptoms such as tremor, rigidity, bradykinesia, postural instability, and gait alterations (2).

In addition, PD presents a broad range of non-motor symptoms, which are equally or even more disabling than motor symptoms, appearing early stages of the disease or even in before diagnosis (3). Some non-motor symptoms are sleep alterations, mood and perception changes, attention and memory problems, cardiovascular and gastrointestinal disorders, fatigue, urinary, and sexual function disorders (3).

Scales have been developed to measure specific aspects of PD, focusing on motor and non-motor symptoms. However, there are less global indices that provide a general view of the patient’s condition (4). A clinimetric index, the *Clinical Impression of Severity Index of Parkinson’s Disease* (CISI-PD) was published in 2006 (4). This index provides information about the degree of severity of the patient which completes and summarizes the data obtained from other scales (5). Moreover, its simplicity and ease of use make it a very helpful tool in both clinical practice and research (5).

The CISI-PD may be considered an appropriate assessment of PD global severity. A recent study has demonstrated that this index performed better than other frequently used global evaluations coming from clinicians (CGIS) and patients (PGIS) (6). In comparison with the Hoehn and Yahr (HY), the CISI-PD correlated at a similar level with other measures of PD severity but offers specific information on four important aspects of the disease and is more precise (range of score: 0–24).

An international and multicenter study carried out to validate the CISI-PD showed that the main cross-sectional determinant factors of disease severity were the patient’s motor condition, disease duration, depression and, to a lesser extent, age and comorbidity (5). However, there is a lack of longitudinal studies using the CISI-PD that would allow finding its main predictors.

To address this deficit, we conducted a longitudinal study to assess how the evaluation of PD global severity, measured by the CISI-PD, changed over time. In addition, we developed a model of global severity to know which motor and non-motor baseline variables could predict PD global severity at 1, 2, and 3 years.

## Materials and Methods

### Design

This was a multicenter, national, observational, and longitudinal study, with a baseline assessment and annual evaluations over 3 years, and a total of four time points. The data collection involved a total of 23 neurologists from 21 hospitals distributed all over Spain.

### Participants

A sample was selected from patients seen consecutively in the neurology or movement disorder departments of various hospitals in Spain. The following inclusion criteria were applied: age equal or above 30 years; idiopathic PD diagnosis carried out by neurologists’ expert in movement disorders, according to the modified criteria of the Brain Bank of the UK Parkinson’s Disease Society (7); and having signed informed consent. Exclusion criteria were the presence of medical or psychiatric comorbidity that would hinder an adequate assessment of PD.

This study was carried out in accordance with the recommendations of Research Ethics Committee of the Carlos III Health Institute and the Clinical Research Ethics Committee of the La Princesa Hospital, Madrid. All subjects gave written informed consent in accordance with the Declaration of Helsinki.

The baseline evaluation included 389 patients with idiopathic PD and 205 completed the follow-up. The causes for case loss at follow-up were as follows: 52 due to inability of researchers to continue; 21 patients refused to remain in the study; 12 patients died; 10 patients moved; 7 patients changed diagnosis; 9 patients dropped out for other reasons (2 could not attend the visits; 2 developed a cognitive impairment; 2 and other comorbidities that prevented follow-up; 2 were impossible to contact; 1 and changed specialist). In 73 cases, there were missing values in some questionnaire items.

### Assessments

All assessment instruments were specific for PD, except for the Hospital Anxiety and Depression Scale (HADS) (8) and the questionnaire EQ-5D with three response levels (EQ-5D-3L) (9), which are generic scales. Sociodemographic and clinical characteristics were also recorded, including levodopa equivalent dose (LED) (9). Table S1 in Supplementary Material summarizes the scales applied in the study.

#### Self-assessment Instruments

The HADS is made of 14 items grouped into two subscales that evaluate the presence of anxiety and depression (8). Items are scored from 0 (no problem) to 3 (severe problem), and a score above 10 in each sub-scale indicates the presence of anxiety or depression, respectively. It can be used in the hospital and community environments, and its validity has been confirmed in patients with PD (10).

The SCales for Outcomes in PArkinson’s disease SCOPA-Sleep (11) is a scale comprising two sections that evaluate night-time sleep (SCOPA-NS) and daytime sleepiness (SCOPA-DS). The SCOPA-Autonomic (SCOPA-AUT) measures autonomic dysfunction in the following areas: gastrointestinal; urinary; cardiovascular; thermoregulatory; pupillomotor; and sexual dysfunction (male or female) (12). It is composed by 25 items, with score ranging from 0 (“never”) to 3 (“often”) and a maximum total score of 69, and the higher score, the worse autonomic dysfunction (13).

The EQ-5D-3L questionnaire is a tool that evaluates health-related quality of life and health status (9). It provides an index whose value may range from 0, representing death, and 1, representing the best health status, and it may have negative values that would represent a state “worse than death.” In addition, the EQ-5D-3L questionnaire contains a visual analog scale (EQ-VAS) assessing current health status. It is a useful scale in patients with PD (14, 15).

#### Instruments Administered by the Neurologist

The SCOPA-Cognition (SCOPA-COG) assesses four cognitive domains that are typically affected by the disease: memory (four items); attention (two items); executive function (three items); and visuospatial function (one item) (16). The maximum total score is 43, and a higher score reflects a better performance (16). The Parkinson’s Psychosis Rating Scale modified (PPRSm) is a scale that assesses the presence of psychotic symptoms in PD and comprises six items. Each item scores from 0 (“absent”) to 3 (“severe”), and the higher score, the more severe the symptoms (17–19). The SCOPA-Motor is a tool that assesses the motor aspects of the disease and contains 21 items grouped in 3 sections (20): motor exploration (SCOPA-Motor EM), with 10 items; ADL (SCOPA-Motor ADL), made up of 7 items; and motor complications (SCOPA-Motor COMP) that contains 4 items. All items score range from 0 (“normal”) to 3 (“severe”), and the higher score, the greater the severity (20).

The CISI-PD is a clinimetric index that reflects the neurologist’s impression of the patient’s severity. It comprises four items (motor signs, disability, motor complications, and cognitive state) that are assessed by the neurologist after interview and clinical exam (4, 5). Each item ranges from 0 (“normal”) to 6 (“severe”); an index is obtained from the sum of these scores, ranging 0–24. Values of 1–7 represent mild disease, 8–14 moderate, and 15 or above severe PD (5).

The HY classification assesses motor alteration and disability in five stages. Stage 1 is mild, with unilateral symptoms and stage 5 is the most severe, when the patient is bedridden or confined to a wheelchair (21).

### Data Analysis

First, we assessed whether there were important differences between individuals with and without missing data at follow-up (22). For this purpose, chi-square and *t*-tests were applied comparing the groups that did and did not complete the study. The difference in the scores between time 0 (baseline) and time 3 (third follow-up), taking only these two time points, was analyzed using paired-sample *t*-test or the Wilcoxon test (depending on whether or not they followed a normal distribution, respectively). Tables 1 and 2 list all variables assessed.

**Table 1:** Descriptive analysis: complete vs. incomplete cases.

	All		Respondents			Non respondents			*t*-Test (*p*-value)
	Mean/%	SD	*N*	Mean/%	SD	*N*	Mean/%	SD	
Gender (%)									
Male	54.24%		108	52.68%		103	55.98%		0.515*
Female	45.76%		97	47.32%		81	44.02%		
Age	65.92	11.15	205	63.71	10.71	184	68.39	11.14	<0.001
Disease duration (years)	8.11	6.00	205	7.67	5.65	184	8.59	6.34	0.131
LED	547.40	371.62	205	493.77	343.67	184	607.16	392.86	0.003
HADS total baseline	13.10	7.60	205	12.56	6.78	184	13.70	8.40	0.147
SCOPA-AUT total baseline	20.45	11.07	205	19.39	10.85	184	21.64	11.21	0.045
SCOPA-COG baseline	23.24	7.36	205	24.99	6.13	184	21.29	8.11	<0.001
CISI-PD Baseline	7.68	4.25	205	7.02	3.95	184	8.43	4.46	0.001
CISI-PD time 1	7.81	4.29	205	7.28	3.95	184	8.73	4.71	0.005
CISI-PD time 2	8.27	4.13	205	7.94	3.83	184	9.45	4.91	0.034
CISI-PD time 3	8.79	3.94	205	8.70	3.96	184	9.61	3.66	0.296

*p-Value of chi-square test.

*CISI-PD, Clinical Impression of Severity Index of Parkinson’s Disease; LED, levodopa equivalent dose; HADS, Hospital Anxiety and Depression Scale; SCOPA-AUT, SCOPA-autonomic scale; SCOPA-COG, SCOPA-Cognition scale*.

*Bonferroni correction: p < 0.003*.

**Table 2:** Changes in clinical variables between baseline and time 3 (*n* = 205).

	Time 0		Time 3		*t*-Test^a^/Wilcoxon^b^ (*p*-value)
	Mean	SD	Mean	SD	
HADS-ANXIETY	7.17	4.02	7.03	4.27	N.S.^a^
HADS-DEPRESSION	5.46	3.62	5.95	3.96	N.S.^a^
HADS total	12.64	6.79	12.99	7.48	N.S.^a^
SCOPA-NS	5.36	4.05	4.59	3.44	N.S.^a^
SCOPA-DS	3.69	3.12	3.77	2.90	N.S.^a^
SCOPA-AUT total	17.83	9.62	20.99	9.83	<0.001^a^
EQ-5D index	0.71	0.25	0.65	0.28	<0.001^a^
EQ-VAS	64.06	19.89	60.22	18.21	N.S.^a^
SCOPA-COG	24.91	6.14	24.50	7.30	N.S.^a^
PPRSm	1.07	1.47	1.24	1.58	N.S.^b^
SCOPA-MOTOR EM	7.38	4.46	8.80	5.10	<0.001^a^
SCOPA-MOTOR ADL	5.00	3.00	6.57	3.89	<0.001^a^
SCOPA-MOTOR COMP	2.09	2.80	2.74	2.70	<0.001^b^
SCOPA-MOTOR total	14.51	7.72	18.05	9.14	<0.001^a^
CISI-PD	6.98	3.91	8.71	3.98	<0.001^a^

*^a^T-Test*.

*^b^Wilcoxon*.

*N.S., non-significant difference; SCOPA-MOTOR EM, SCOPA-motor exploration scale; SCOPA-MOTOR ADL, SCOPA scale on activities of daily life; SCOPA-MOTOR COMP, SCOPA-motor complications scale; SCOPA-MOTOR Total, total motor score; SCOPA-NS, SCOPA night-time sleep scale; SCOPA-DS, SCOPA daytime sleepiness scale; SCOPA-AUT Total, total score of SCOPA scale of autonomic symptomatology; SCOPA-COG, SCOPA Cognitive scale; CISI-PD, Clinical Impression of Severity Index of Parkinson’s Disease; HADS, Hospital Anxiety and Depression Scale; PPRSm, Parkinson’s Psychosis rating Scale modified; EQ-5D Index, index of EQ-5D Quality of Life questionnaire; EQ-VAS, analog visual scale of EQ-5D*.

The changes in CISI-PD scores were studied with analysis of variance for repeated measures, taking time as a factor. All previous analysis were adjusted for multiple comparisons using the Bonferroni method (statistical significance *p* < 0.003). In addition, the effect size (d) was calculated to describe the magnitude of change of the CISI-PD throughout the study period. It was defined as the mean difference divided by the SD of baseline scores. In accordance with Cohen’s criteria, the magnitude of change was considered small if, when expressed as an absolute value, it fell between 0.2 ≤ *d* < 0.5; moderate, 0.5 ≤ *d* < 0.8; and large, *d* ≥ 0.8 (23).

To analyze the individual change in PD global severity (CISI-PD score), and taking into consideration the two-level hierarchical structure of the data, we performed a multilevel linear regression with random effects. Level 1 was made up of repeated measures and described each individual’s evolution over time, based on the following categories: T0 = baseline; T1 = 1 year; T2 = 2 years; T3 = 3 years of follow-up. Level 2, made up of individuals, described the change in the trajectories between individuals and identified factors, which explain the differences between them (24). The first step entailed performance of a non-conditional or empty multilevel model, which contained no predictors. The second step involved the creation of a predictive model including the following variables measured at baseline: global severity status of the disease (CISI-PD), neuro-psychiatric measurements (HADS Total, PPRSm, SCOPA-COG, SCOPA-Sleep), psychosocial measurements (EQ-5D-3L index), sociodemographic variables (age and sex), and clinical variables (disease duration; LED; EQ-VAS; SCOPA-Motor EM items bradykinesia, rigidity, tremor, and axial; and SCOPA-AUT). Since there were only three separate follow-up points in time, time was introduced in the model as a discrete variable.

We calculated the intraclass correlation coefficient (ICC) for both the empty and the predictive model, providing information on the proportion of variance due to differences across individuals. A stepwise backward regression (criterion: *p* < 0.05) was applied to remove the non-significant variables from the predictive model to get a model that meets the principle of parsimony, while ensuring absence of collinearity. This regression model presented 30.30% of losses in the outcome variable and 3.12% in the rest of variables with missing values. Missing data on CISI-PD at each of the follow-up time point was: time 1, 17.2%; time 2, 32.4%; and time 3, 41.4%.

To deal with the possible bias resulting from patients lost to follow-up, data imputation was used on 33.42% of the cases (25). Due to the missing values that were found in the outcome variable (CISI-PD at follow-up), a “multiple imputation, then deletion” (MID) procedure was developed (26, 27). In this method, the cases with imputed outcome variable are excluded. In addition, a sensitivity analysis was performed to assess the suitability of the imputation models. For this purpose, MID was compared with a model with missing values and a multiple imputation by chained equations, expecting similar results in the three models.

The predictions were obtained through Monte Carlo simulation. Also, the mean absolute error and the mean square error between observed and predicted values were calculated, using the same sample size, to measure the accuracy of the models. The adjusted coefficient of determination (adjusted *R*^2^) assessed the fit of the data to the statistical analysis. We also compared the predicted and observed values through visual inspection of the scatter-plot for the three time points.

Finally, we calculated the estimated means of CISI-PD in the time points and observed the linear trend through an analysis of co-variance for repeated measures, adjusting for the variables selected in the multilevel stepwise regression.

Table S2 in Supplementary Material summarizes the steps followed in data analysis. The SPSS version 22 was used for data analysis and Stata version 14 for predictive and multiple imputation models.

## Results

In the baseline sample (*n* = 389), 54.20% (*n* = 211) of patients were male, the mean age was 65.92 (SD: 11.15) years, and 80.50% (*n* = 313) were married. The mean years of education were 10.06 (SD: 5.63), and 54% (*n* = 208) were retired. The average PD duration was 8.11 (SD: 6.00) years. About 5.9% of patients (*n* = 23) were treated with only levodopa, 20.4% (*n* = 79) with a dopamine agonist and 7.06% (*n* = 29) took both; the mean of LED was 547.4 (SD: 371.62). The median HY staging was 2, and the distribution of the sample was as follows: stage 1, 25.20% (*n* = 97); stage 2, 50.10% (*n* = 193); stage 3, 19.50% (*n* = 75); stage 4, 4.70% (*n* = 18); and stage 5, 0.50% (*n* = 2).

There were significant differences in age, baseline CISI-PD, and SCOPA-COG values between patients with complete or missing values (Table 1). Table 2 shows the changes in the clinical variables from baseline to time 3 experienced by the group that completed the study. The CISI-PD effect size between times 0 and 3 was 0.44.

The non-conditional or empty multilevel model of the CISI-PD dependent variable showed an ICC of 0.816. The MID model (Table 3) was established to estimate the disease global severity (as expressed by the CISI-PD) in times 1, 2, and 3. In addition to CISI-PD at baseline, the following baseline measures were statistical significant CISI-PD predictors: HADS, SCOPA-AUT, and SCOPA-COG. Disease duration, PPRSm, SCOPA-Sleep, EQ-VAS, EQ-5D index, SCOPA-Motor EM items bradykinesia, rigidity, tremor, and axial, age, and sex did not reach statistical significance.

**Table 3:** Predictive models to estimate the patient’s degree of severity (expressed by the CISI-PD) at times 1, 2, and 3, from baseline values.

	Regression model with no missing data (*n* = 792)					Multiple imputation by chained equations (*n* = 1,167)					Multiple imputation, then deletion (*n* = 813)				
	β	SE	*p*-Value	95% CI		β	SE	*p*-Value	95% CI		β	SE	*p*-Value	95% CI	
**Time (reference 1)**															
Time 2	0.621	0.157	<0.001	0.313	0.928	0.639	0.195	0.002	0.250	1.028	0.648	0.156	<0.001	0.341	0.955
Time 3	1.389	0.165	<0.001	1.065	1.712	1.316	0.257	<0.001	0.756	1.875	1.405	0.165	<0.001	1.081	1.729
LED baseline	0.002	0.001	<0.001	0.001	0.003	0.001	0.001	0.001	0.000	0.002	0.002	0.001	<0.001	0.001	0.003
HADS baseline	0.046	0.021	0.034	0.004	0.088	0.047	0.020	0.027	0.006	0.089	0.047	0.021	0.027	0.005	0.088
SCOPA-AUT total baseline	0.040	0.015	0.007	0.011	0.069	0.043	0.012	<0.001	0.020	0.067	0.043	0.015	0.003	0.014	0.071
SCOPA-COG baseline	−0.115	0.021	<0.001	−0.155	−0.075	−0.104	0.018	<0.001	−0.141	−0.068	−0.114	0.021	<0.001	−0.156	−0.072
CISI-PD baseline	0.530	0.041	<0.001	0.449	0.611	0.538	0.033	<0.001	0.472	0.604	0.523	0.040	<0.001	0.444	0.602
Constant	4.277	0.677	<0.001	2.951	5.603	4.249	0.647	<0.001	2.928	5.570	4.269	0.690	<0.001	2.913	5.624
MAE	2.100					2.115					2.103				
MSE	7.208					7.287					7.210				
Adjusted *R*^2^	0.525					0.517					0.526				

*β, coefficient of the regression model; 95% CI, confidence interval at 95% level; CISI-PD, Clinical Impression of Severity Index of Parkinson’s Disease; LED, levodopa equivalent dose; HADS, Hospital Anxiety and Depression Scale; SCOPA-AUT, SCOPA-autonomic scale; SCOPA-COG, SCOPA-Cognition scale; MAE, mean absolute error; MSE, mean square error; Adjusted R^2^, adjusted coefficient of determination*.

The following formula describes the prediction model:

CISI-PDtime i= Time2×0.648 + Time3×1.405 + LED×0.002 + HADS baseline×0.047+SCOPA AUT Baseline×0.043−SCOPA COG Baseline×0.114 + CISI PD Baseline×0.523 + 4.684.

This equation may be used to estimate the CISI-PD value of a person at follow-up. Take the example of a patient with a value of 550 of LED, HADS value of 13, SCOPA-AUT of 20, SCOPA-COG of 25, and CISI-PD at baseline of 8. The value of CISI-PD, after 1 year would be 8.589 (CISI-PD_time 1_ = 0 × 0.648 + 0 × 1.405 + 550 × 0.002 + 13 × 0.047 + 20 × 0.043 − 25 × 0.114 + 8 × 0.523 + 4.684); at 2 years 9.237 (CISI-PD_time 2_ = 1 × 0.648 + 0 × 1.405 + 550 × 0.002 + 13 × 0.047 + 20 × 0.043 − 25 × 0.114 + 8 × 0.523 + 4.684); and at 3 years 9.994 (CISI-PD_time 3_ = 0 × 0.648 + 1 × 1.405 + 550 × 0.002 + 13 × 0.047 + 20 × 0.043 − 25 × 0.114 + 8 × 0.523 + 4.684).

**Figure 1:**
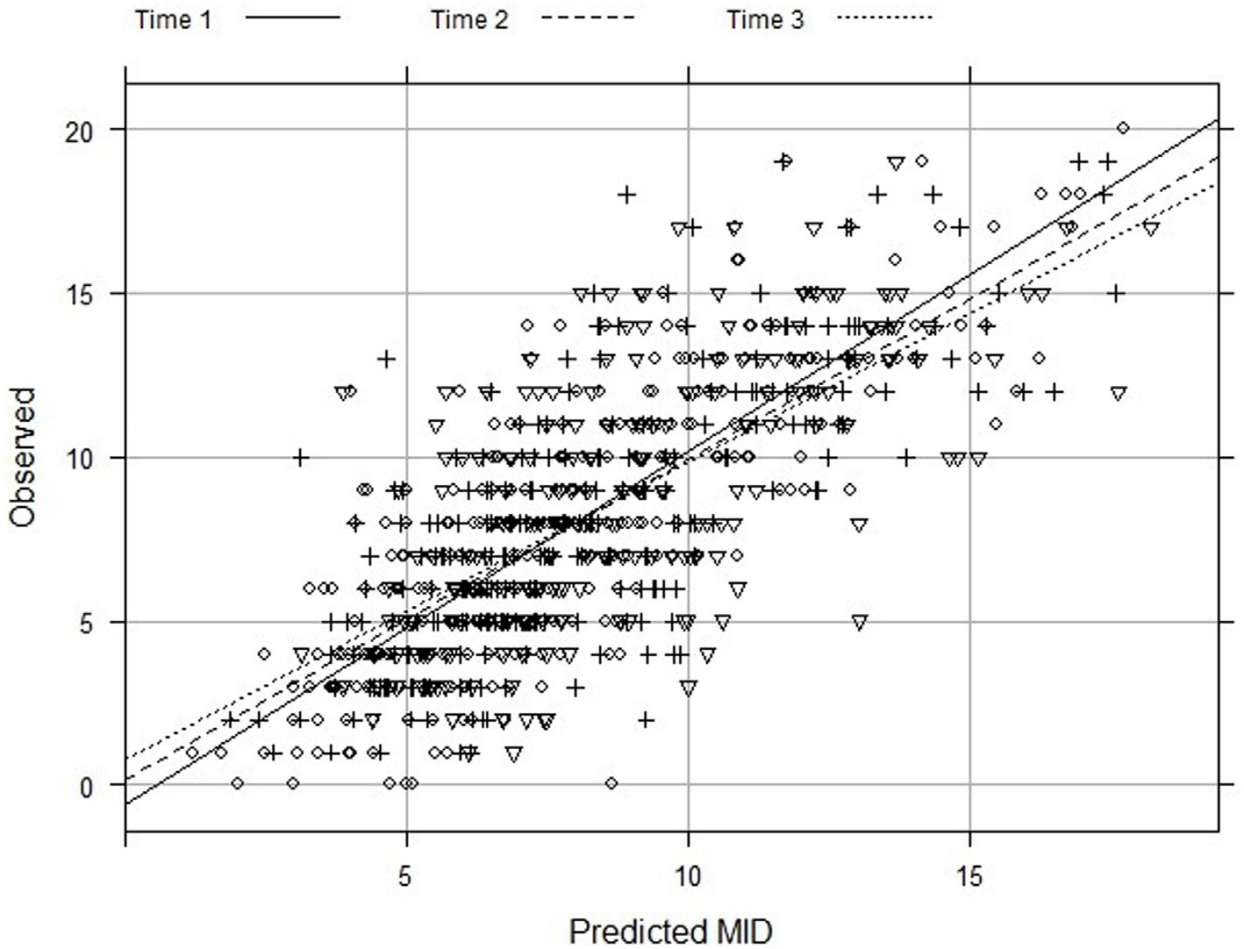
Observed and predicted values of Clinical Impression of Severity Index of Parkinson’s Disease from multilevel regression with multiple imputation, then deletion (MID).

When the covariables described in this formula were entered into the empty model, the ICC was 0.563. All models showed comparable results in the sensitive analysis (Table 3). Figure 1 shows similar results when comparing the prediction of the model and the data obtained, especially for CISI-PD values ranging 5–10 (mild to moderate).

**Figure 2:**
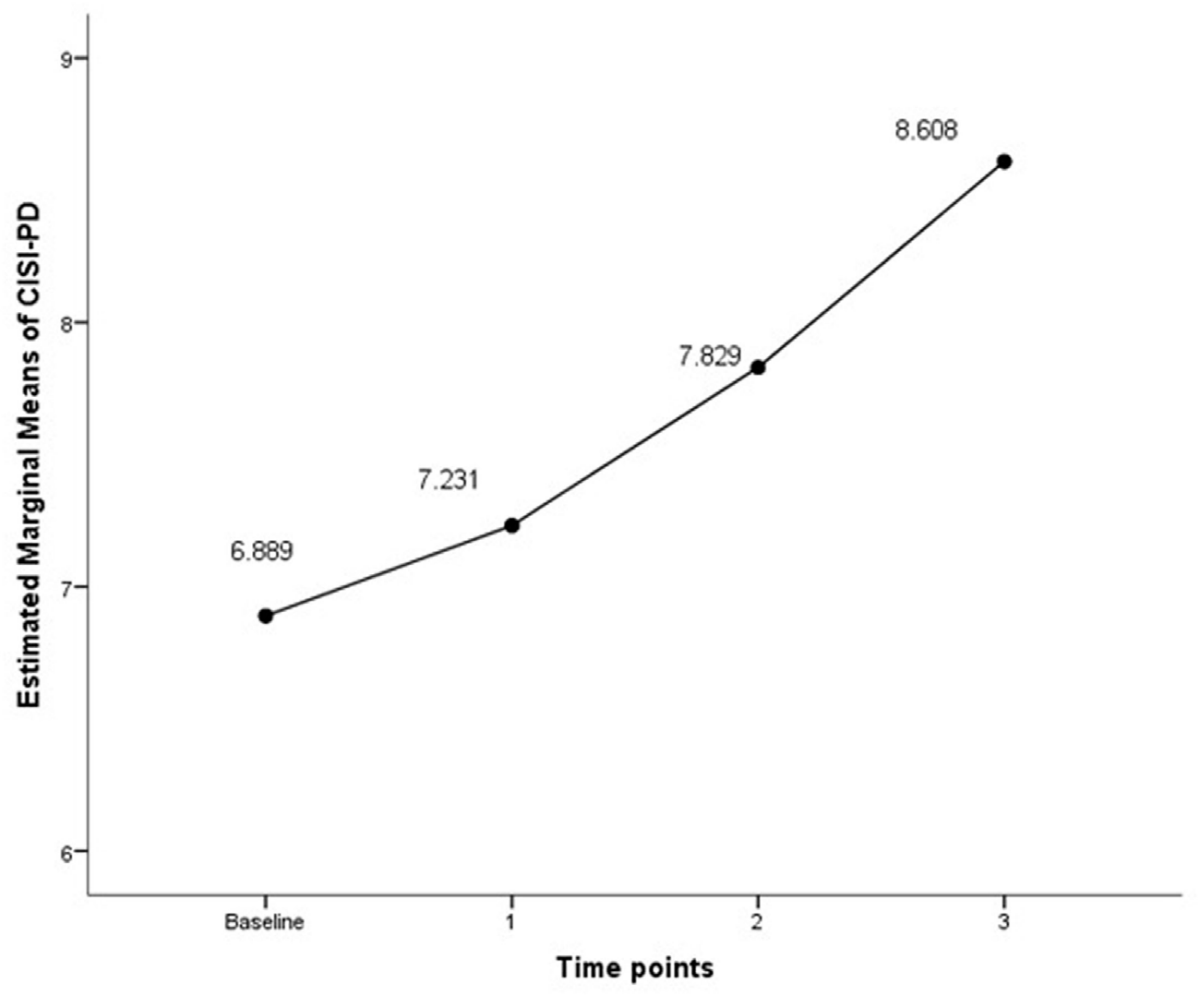
CISI-PD scores throughout the follow-up (3 years). Covariates at baseline appearing in the model are evaluated at the following values: LED = 496.77, HADS = 12.47, SCOPA-AUT = 19.24, and SCOPA-COG = 25.07. CISI-PD, Clinical Impression of Severity Index of Parkinson’s Disease; LED, levodopa equivalent dose; HADS, Hospital Anxiety and Depression Scale; SCOPA-AUT, SCOPA-autonomic scale; SCOPA-COG, SCOPA-Cognition scale. Significant linearity test (*F* statistic = 11.176, *p*-value = 0.001).

The estimated means of CISI-PD scores increased significantly between all yearly assessments (*p* < 0.001), following a significant linear trend (Figure 2).

## Discussion

The CISI-PD is a measure of global PD severity; it is easy to apply, valid, reliable, accurate, and useful in both clinical practice and research. It provides a comprehensive assessment of important manifestations of PD not included in other global rating scales, for example, HY staging (4). The main objective of this study was to design a predictive model of the global severity of PD over 3 years using the CISI-PD, based on the clinical data obtained at baseline from a sample of patients with idiopathic PD. The model can complement knowledge about the evolution of PD progression over 3 years.

Our study showed that at the baseline, patients who completed the study were younger, with better baseline CISI-PD and cognitive function. These characteristics were similar to those of patients in another longitudinal study (28). To account for the sample loss, data imputation was used, with good results.

A PD global severity (CISI-PD) worsening of small magnitude was observed over the 3-year follow-up. On the one hand, this small experienced deterioration may be due to the fact that PD worsening is slowed by the effect of anti-Parkinsonian medication (2, 29). On the other hand, it might indicate that the follow-up period was not long enough to detect larger changes in a disease with a slow and long progression.

In a previous study, the main cross-sectional determinants of CISI-PD were motor status (SCOPA-Motor Total), disease duration, depression and, to a lesser extent, age and comorbidity (5). In our longitudinal study, baseline values of PD global severity (CISI-PD), depression and anxiety symptoms (HADS), cognitive function (SCOPA-COG), autonomic symptoms (SCOPA-AUT), and LED were predictors of CISI-PD at 1, 2, and 3 years. A different study design (cress-sectional vs. longitudinal) might explain the differences found in the factors associated to CISI-PD in both studies.

Several studies highlighted the importance of non-motor symptoms, such as cognitive impairment and autonomic dysfunction, in PD severity and progression. One study concluded that PD was associated with significantly higher morbidity rates associated with conditions such as mental and psychiatric, nervous system, gastrointestinal, genitourinary problems, among others (30). The presence of cognitive impairment was also associated with increased mortality (31) and a longer duration of disease increased the prevalence of dementia (32).

According to our results, the presence of the treatment as a predictor indicates its importance in Parkinson’s disease clinical management (2, 33). In early stages of the disease, patients may not need anti-Parkinsonian treatment. In fact, although the l-DOPA is the most effective for the symptomatic treatment of PD, the presence of motor complications associated with this treatment, such as motor fluctuations and dyskinesias, is a limitation that has led some physicians to advocate that the initiation of this treatment must be delayed (34). However, this treatment is necessary as PD progresses, which may partly account that its presence is associated with a higher score overall severity of PD.

The non-conditional or empty multilevel model of the CISI-PD yielded an ICC of 0.816. In other words, 81.6% of the variability observed in the CISI-PD was attributable to differences between individuals at baseline, and the remaining 18.4% was due to each individual’s evolution over time. The ICC dropped to 0.563 when the predictive model was adjusted by the significant covariables, which means that the 56.3% of the observed variability was attributable to differences between individuals at baseline and the remaining 43.7% was due to each individual’s evolution over time. These results imply that the disease progression depends more on the individual’s baseline characteristics than on the passage of time. Previous studies suggest that the rate of progression of PD symptoms and signs vary widely between different patients, according to their baseline characteristics (31). For instance, the presence of cognitive impairment at baseline has been associated with a faster progression of motor impairment (35).

The assessment of our results is subject to a number of limitations. First, a loss of almost half of the patients over a period of only 3 years may result in less precise estimates. However, bias was minimized with the use of multiple imputation techniques, with good results in sensitivity analysis (36). Second, the development of new concomitant diseases, expected in older age groups, was not taken into account. The third limitation is the relative short length of follow-up for a chronic condition as PD, which in our case lasted 3 years. Taken into account that some PD symptoms may appear later, new longitudinal studies over extended time span would be necessary (37). Fourth, we did not apply the predictive model in a different sample of our study, so we do not know how good are the predictions under real live conditions and in what extent the predictions could be superior to expert assumptions. For this reason, it would be appropriate to design new longitudinal prospective studies with a similar follow-up and compare the results of the assessments of each time point and the assumptions of the experts with those obtained with the predictive model. In addition, other studies could be carried out using different endpoints focused, for instance, on non-motor complications such as cognitive impairment or impulse control disorders.

Despite such limitations, our results identify factors that influence PD progression. This might be of great value for clinicians for planning, implementation, and evaluation of intervention strategies to promote comprehensive rehabilitation and social inclusion of people with PD.

From our study, we can conclude that after the 3-year follow-up, a relatively short period, there was a PD global severity worsening of small magnitude. This study also showed that the baseline values of the overall degree of PD severity, cognitive function, depression and anxiety symptoms, autonomic symptoms, and LED were significant predictors of overall severity of PD at 1, 2, and 3 years. Our results can help toward a better understanding of the disease evolution in a short, 3-year period of PD global severity and to understand the role of non-motor symptoms such as depression, anxiety, autonomic, and cognitive symptoms, in the progression of the disease (30–32, 37).

## ELEP Project Members

ELEP (Longitudinal Study of PD) project members are as follows:

**M. Aguilar and P. Quilez-Ferrer** (Mutua de Terrassa, Barcelona); **M. Alvarez Sauco** (Elche General Hospital, Alicante); **A. Bayes** (Teknon Clinic, Barcelona); **M. Blazquez** (Asturias Central Hospital, Oviedo); **A. Bergareche** (Bidasoa Hospital, Guipuzcoa); **V. Campos** (Xanit International Hospital, Benalmadena, Malaga); **M. Carballo** (Virgen del Rocío Hospital, Sevilla); **M. J. Catalan** (San Carlos Hospital, Madrid); **J. Chacon** (Virgen de la Macarena Hospital, Sevilla); **E. Cubo** (Burgos Hospital); **J. M. Fernandez-Garcia** (Basurto Hospital, Bilbao); **B. Frades-Payo** (CIEN Foundation, Carlos III Institute of Health, Madrid); **P. Garcia-Ruiz-Espiga** (Jimenez-Diaz Foundation Hospital, Madrid); **J. Kulisevsky** (Sant Pau Hospital, CIBERNED, Barcelona); **G. Linazasoro** (Gipuzkoa Polyclinic, San Sebastian);**J. Lopez del Val** (Lozano Blesa Hospital, Zaragoza);**J. C. Martinez-Castrillo** (Ramón y Cajal Hospital, Madrid); **P. Martinez-Martin, M. J. Forjaz, and C. Rodriguez-Blazquez** (Carlos III Institute of Health, Madrid); **A. Mendoza** (Segovia Hospital); **P. Mir** (Virgen del Rocio Hospital, CIBERNED, Sevilla); **I. Posada** (Doce de Octubre Hospital, CIBERNED, Madrid);**F. Valldeoriola** (Clinical Hospital, CIBERNED, Barcelona); **L. Vela** (Alcorcon Hospital Foundation, Madrid); and **F. Vivancos and I. Ybot** (La Paz Hospital, Madrid).

## Ethics Statement

This study was carried out in accordance with the recommendations of Research Ethics Committee of the Carlos III Health Institute and the Clinical Research Ethics Committee of the La Princesa Hospital, Madrid with written informed consent from all subjects. All subjects gave written informed consent in accordance with the Declaration of Helsinki.

## Author Contributions

PM-M and MJF conceived and designed the study. AA, JMT-J, and J-MR-A run the statistical analyses. AA and JMT-J wrote the first drafts of the paper. All the authors including CRB, reviewed and critiqued the manuscript. All the authors were responsible for the decision to submit the manuscript for publication.

## Disclaimer

This study presents independent results and research. The views expressed are those of the authors and not necessarily those of the Instituto de Salud Carlos III.

## Conflict of Interest Statement

JMT-J, MJF, AA, CR-B, and J-MR-A report no disclosures. PM-M received honoraria from speaking engagements at scientific meetings of Italfarmaco and Teva, and from serving in a scientific advisory board of AbbVie; grants from Carlos III Institute of Health [FIS], Reina Sofia Foundation, and Michael J. Fox Foundation; and his employment is at Carlos III Institute of Health.
