# Supplementary material for: Parkinson’s Disease Severity at 3 Years Can Be Predicted from Non-Motor Symptoms at Baseline
Source: Front Neurol. 2017 Oct 30;8:551. doi: 10.3389/fneur.2017.00551 (PMC5674937; doi:10.3389/fneur.2017.00551)
Supplement: Supplementary file 1 [file data_sheet_1.docx]

**Supplemental Table 1: Scales applied in the study.**

| **Abbreviation** | **Full name** | **Reference** | **Construct measured** | **Number of items** | **Response scale** |
| --- | --- | --- | --- | --- | --- |
| HADS | Hospital Anxiety and Depression Scale | 7,10 | Anxiety and depression | 7+7 | 0-3 |
| SCOPA-SLEEP | SCales for Outcomes in PArkinson's disease - sleep | 11 | Night-time sleep (SCOPA NS) and daytime sleepiness (SCOPA DS) | 5+6 | 0-3 |
| SCOPA-AUT | SCales for Outcomes in PArkinson's – Autonomic Dysfunction | 12, 13 | Autonomic dysfunction in the following areas: gastrointestinal; urinary; cardiovascular; thermoregulatory; pupillomotor and sexual dysfunction (male or female) | 25 | 0-3 |
| EQ-5D-3L | EQ-5D, 3 levels | 8, 14, 15 | Quality of life and health status | 5 + visual analogue scale | 1-3; 0-100 |
| SCOPA-COG | SCales for Outcomes in PArkinson's –Cognition | 16 | Cognitive domains: : memory (four items); attention (two items); executive function (three items); and visuospatial function (one item) | 10 | 0 to 2-6 |
| PPRSm | Parkinson’s Psychosis Rating Scale modified | 17-19 | Psychiatric symptoms | 30 | 0-3 |
| SCOPA-Motor | SCales for Outcomes in PArkinson's –Motor | 20 | Motor aspects: motor exploration (SCOPA-Motor EM); ADL (SCOPA-Motor ADL); and motor complications (SCOPA-Motor COMP) | 10+7+4 | 0-3 |
| Clinical Impression of Severity Index of Parkinson’s Disease | CISI-PD | 4 | Parkinson’s disease severity | 4 | 0-6 |
| Hoehn & Yahr staging | HY | 21 | motor alteration and disability | 1 | 1-5 |

Note: Reference numbers correspond to those cited in the text.

**Supplemental Table 2. Steps followed in data analysis.**

| **Step** | **Statistical test** | **Rational** | **Results table/figure** |
| --- | --- | --- | --- |
| 1. Differences between individuals with and without missing data | Chi-square test and t-test | In the 3-year follow-up, several participants dropped out, leading to missing data. This analysis if the final sample is very different form the initial one. | Table 1 |
| 1. Score difference between Time 0 (baseline) and Time 3 (third follow-up), taking only these two time points | Paired-sample t-test and Wilcoxon test | Provides a global description of change over time. | Table 2 |
| 1. Change on CISI-PD over time, in the 4 time points | Analysis of variance (ANOVA), taking time as a within subjects factor; effect size d | This answers the first goal of the study at a bivariate level: to assess how the evaluation of PD global severity, measured by the CISI-PD, changed over time. |  |
| 1. Analysis of the individual change in PD global severity (CISI-PD score) | Multi-level random effects linear regression. Level 1: individual’s evolution over time, T0= baseline, T1= 1 year, T2= 2 years, T3= 3 years of follow-up; level 2: individuals | Multivariate analysis of the individual change in PD global severity (CISI-PD score) and associated factors. | Table 3 |
| 1. Data imputation | Multiple imputation, then deletion (MID) | Replacement of missing data with estimated values. | Table 3 |
| 1. Sensitivity analysis | MID were compared to a model with missing values and a multiple imputation by chained equations (MICE) | Assess the suitability of the imputation models. | Table 3 |
| 1. Validation of predictive model | Calculation of mean absolute error (MAE), mean square error (MSE) and adjusted R^2^ | To assess the goodness of fit of the final predictive model. | 3 last lines of Table 3; Figure 1 |
| 1. Test of the linear trend: change of CISI-PD total mean scores by time. | Analysis of co-variance (ANCOVA), adjusting for the variables selected in the multilevel stepwise regression | Predictive model of global severity to know which motor and non-motor baseline variables could predict PD global severity at 1, 2 and 3 years. | Figure 2 |
